# Deep Learning-Based Segmentation and Volume Calculation of Pediatric Lymphoma on Contrast-Enhanced Computed Tomographies

**DOI:** 10.3390/jpm13020184

**Published:** 2023-01-20

**Authors:** Michał Klimont, Agnieszka Oronowicz-Jaśkowiak, Mateusz Flieger, Jacek Rzeszutek, Robert Juszkat, Katarzyna Jończyk-Potoczna

**Affiliations:** 1 Department of Radiology, Poznań University of Medical Sciences, Dluga 1/2, 61-848 Poznan, Poland; 2 Fast-Radiology, Poland; 3 Department of Pediatric Radiology, Institute of Pediatrics, Poznań University of Medical Sciences, Szpitalna 27/33, 60-572 Poznan, Poland; 4 1st Department of Radiology, National Institute of Oncology, W.K. Roentgena 5, 02-781 Warsaw, Poland

**Keywords:** nnU-Net, deep learning, pediatric lymphoma, computed tomography, segmentation

## Abstract

Lymphomas are the ninth most common malignant neoplasms as of 2020 and the most common blood malignancies in the developed world. There are multiple approaches to lymphoma staging and monitoring, but all of the currently available ones, generally based either on 2-dimensional measurements performed on CT scans or metabolic assessment on FDG PET/CT, have some disadvantages, including high inter- and intraobserver variability and lack of clear cut-off points. The aim of this paper was to present a novel approach to fully automated segmentation of thoracic lymphoma in pediatric patients. Manual segmentations of 30 CT scans from 30 different were prepared by the authors. nnU-Net, an open-source deep learning-based segmentation method, was used for the automatic segmentation. The highest Dice score achieved by the model was 0.81 (SD = 0.17) on the test set, which proves the potential feasibility of the method, albeit it must be underlined that studies on larger datasets and featuring external validation are required. The trained model, along with training and test data, is shared publicly to facilitate further research on the topic.

## 1. Introduction

Lymphomas are the most common blood malignancies in the developed world [1]. The two main categories of lymphomas are non-Hodgkin lymphomas (NHL) and Hodgkin lymphomas (HL) [1]. Worldwide, lymphomas are the ninth most common malignant neoplasms as of 2020 and were diagnosed in 627,439 persons, and caused 283,169 deaths [2].

Standardized staging and response criteria are key to successfully managing patients; what is more, they are essential to compare results and endpoints between studies when comparing treatment efficacy in a population.

There are multiple staging systems for both HL and NHL based on various criteria, including the anatomic disease extent and involvement of extra-nodal sites, as well as clinical and biochemical parameters [3]. The classification that is most commonly used in clinical practice is the Lugano staging classification, introduced in 2011 [4]. Factors taken into account include the number of lymph node regions involved, the presence of the disease on one or both sides of the diaphragm, the involvement of extranodal organs, the presence of systemic symptoms, and the presence of a bulky manifestation [4]. Evaluation of response, according to the Lugano classification, can be based on computed tomography (CT) alone or 18F-fluorodeoxyglucose (FDG) positron emission tomography/computed tomography (PET/CT) [4]. In PET/CT FDG, uptake is commonly assessed against regions of increased physiological activity using the Deauville score [5] and compared to baseline; the disease is then classified as complete, partial, or no metabolic response. In the Lugano criteria for FDG PET/CT, the volume of the metabolically active tumor tissue is not taken into account. CT assessment, on the other hand, is based on two-dimensional tumor measurement of up to six target lesions [4]. Tumor dimensions of each lymph node are multiplied, and these values are then added for all lesions and compared with the baseline [4]. Therefore, it can be argued that the value that is received is proportional to the surface area in the axial plane, which, in turn, reflects the volume of the lesion. The sum of the product for selected target lesions should be an approximate reflection of total tumor volume; however, it may be argued that it is less intuitive than volume for a human reader.

While relatively straightforward and easily applicable, the Lugano response criteria were primarily based on expert opinion, and their development has not been supported by large-scale data analysis [6]. What is more, the criteria for computed tomography restaging are elaborate, and the measurements are difficult to replicate, which results in a relatively high inter- and intra-observer variability [7]. Criteria based on FDG-avidity, on the other hand, are seen as controversial in lymphomas with variable FDG avidity, such as marginal zone lymphomas [4]. Additionally, in some cases, FDG-avid areas constituted only a small portion of the tumor mass (approximately 25%), and that monitoring based on FDG-avid areas potentially limits the prediction of the treatment sensitivity of the whole tumor mass to this small region [8]. In spite of those pitfalls, many lymphoma clinical trials continue to use the Lugano criteria as the best option that is available, albeit many introduce some modifications [9,10].

Because of these challenges in using Lugano and other classifications for staging, restaging, and follow-up of lymphomas, current guidelines recommend the use of FDG PET/CT [11], with the standardized glucose uptake value (SUV), used as a threshold to determine the metabolic tumor volume (MTV), defined as a total tumor volume demonstrating high glucose metabolism [12].

The use of a volume metric has numerous advantages over two-dimensional measurements, as proposed in the Lugano criteria. It eliminates the need for a subjective selection of a lesion and determining the best way of measuring it and, in the case of multiple tumors, the tedious process of identifying and comparing previously selected lesions. It can be hypothesized that using automatically determined tumor volume as a metric could result in a higher accuracy and a lower reading time compared to other methods and measurements performed by human readers. Indeed, it has been shown that PET/CT pretherapy metabolic tumor volume may be an independent prognostic factor in patients with some types of lymphoma, for example, large B-cell lymphoma [13]. It has also been shown that PET/CT can be used in both HL and NHL for monitoring the disease, although there is currently no consensus on cut-off points for both volume [3] and SUV threshold [14], and the positive influence of altering treatment on the basis of PET/CT results alone is not clear [3].

However, it is not always practical to perform PET/CT as a lymphoma follow-up. As mentioned above, some less common lymphoma types have varying FDG avidity, although pediatric lymphomas are more often FDG-avid than adult lymphomas. More importantly, PET/CT examinations may result in increased radiation exposure when compared to a stand-alone CT examination, as the effective dose is a combination of the dose from PET and CT [15], although it is worth noting that new hybrid PET/low dose CT scans do not have to translate to a higher radiation dose than CT. This is especially important when multiple follow-up examinations are required. What is more, PET/CT examination is significantly more expensive and less available, and the use of this examination for a regular follow-up for the sake of a somewhat better accuracy cannot be justified in many cases.

As shown above, both methods based on two-dimensional measurements taken by a radiologist or automatically computed MTV can be useful for staging and follow-up, but they have numerous limitations. From a practical standpoint, it seems that it would be ideal to have a method that would be cheaper and expose patients to less radiation than FDG PET/CT while at the same time being less prone to inter- and intra-observer variability than two-dimensional measurements taken subjectively by a radiologist on a regular CT scan. An alternative use of whole-body MRI is proposed; however, its application seems to be focused more on the initial staging, as diffusion-weighted imaging rather than facilitates finding suspected lymph nodes, than it helps in the treatment evaluation [16]. Thus, it seems appropriate to develop a volume based approach that would be based on CT scans.

It could be argued that calculating volume on plain CT scans does not take into account the metabolic state of the mass and may therefore be less reliable. However, it has been shown that the evolution of MTV and total tumor volume during treatment is similar for HL and that both FDG PET/CT avid and non-avid areas shrink at a similar rate [8].

With all the challenges described above, researchers are searching for alternative methods of volume measurements for lymphoma. To this date, researchers mostly focused their approach on a search for quick and precise estimates of the volume performed by the reporting radiologist that was not computer-aided [17]. However, with the advancement in machine learning, segmentation tasks in medical imaging can now be approached with fully automated methods. There are already solutions developed for anatomical structures segmentation, such as colon [18], lung [19], or even more robust multi-organ segmentation, such as the work by Wasserthal et al., which focused on segmenting 104 anatomical structures [20]. To our knowledge, automated volume calculation based on machine learning has not been used to calculate the volume of chest lymphoma.

The aim of this paper is to present a solution based on machine learning for automatic segmentation and volume calculation of chest lymphoma. Such a tool could theoretically assist both radiologists and clinicians in staging and follow-up of lymphoma in a more objective way than currently available methods. However, it should be emphasized that volume is one of many prognostic and staging factors. Importantly, the aim of this paper is also to release the dataset of segmented pediatric lymphomas for other researchers.

## 2. Materials and Methods

In total, 30 CT scans from 30 different pediatric patients diagnosed with any type of lymphoma were collected. Five patients were randomly selected as a test set and the rest of the cases were used for training and validation. All patients were hospitalized at Karol Jonscher University Hospital, Poznan, Poland between the years 2013 and 2020. Only one CT scan per patient was included to maximize potential variance in the data and increase model generalizability. Scans from the following two CT scanners were used: Siemens SOMATOM Definition AS+ and Siemens SOMATOM Force (Siemens Healthineers, Erlangen, Germany). In all cases, Visipaque (GE Healthcare, Chicago, IL, USA) was used as the contrast agent. The dosage of the contrast agent was calculated using the following formula: for children under 35 kg, a dose of 1 mL per kg of body weight was used; for children over 35 kg, a dose of 40–50 mL was used. The injection rate was between 1.5 and 2.5 mL/s. All scans were performed in portal venous phase.

**Figure 1:**
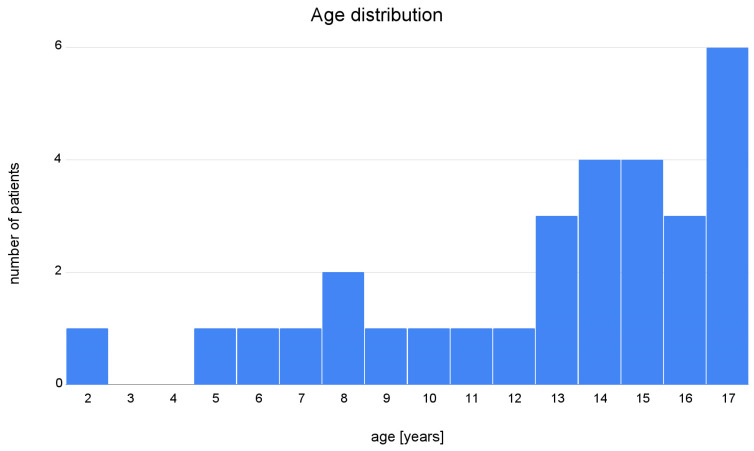
Age distribution of the patients included in the training and testing dataset.

There were 17 boys and 13 girls in the study group, and the mean age was 12.8 (SD 4.1, minimum age 2, maximum age 17). Patients’ age distribution is demonstrated in Figure 1. Median scan size was 296 × 512 × 512 voxels. Full dataset is publicly available at [21], model achieving the best Dice score is available for download at [22], and code for inference is available at [23].

The study, as well as making the scanning data publicly available, was approved by the local Bioethics Committee and the head of the Radiology Department. Furthermore, the patients and/-or their legal guardians consented to the data being used for retrospective research purposes. All data were fully anonymized.

Subsequently, two radiology residents manually segmented the thoracic lymphoma manifestations in 3D Slicer [24]. While performing the segmentations, multiple challenges were encountered, and it was attempted to create a set of rules about how to determine whether or not to include a lesion in the segmentation (Table 1).

To perform automated segmentations, we decided to explore the feasibility of applying deep learning. First developed by Ronneberger et al. in 2015 [25], the U-Net architecture has become a popular approach to segmentation tasks. Its impressive, robust performance has encouraged many researchers to develop modifications, further improving the results [26]. This has led to the development of a variety of new configurations (e.g., extending from two-dimensional to three-dimensional input), however, the parameters were generally preselected for a specific task and not generalizing well to every other problem. This makes parameter selection a time- and resource-consuming task. In 2018, Fabian Isensee et al. [27] developed nnU-Net (‘no-new-UNet’), which is a self-adapting framework that attempts to automatically prepare a well-performing configuration based on specific dataset properties. Not only does it automatically select certain parameters for the researcher, but it also streamlines the research process by providing tools for preparing and performing cross-validation, obtaining predictions for validation set, determining whether to apply post-processing, and deciding upon which, model (2-dimensional, 3-dimensional, 3-dimensional “cascade”, or a combination of them-i.e., ensemble), performs best. It is a popular approach to segmentation in biomedical tasks [26], and considering its simplicity and robustness, serves as a good reference baseline.

Great generalizability of nnU-Net across diverse datasets was demonstrated by Isensee et al. [27]. The framework was developed on 53 various segmentation tasks. Input data modalities included magnetic resonance imaging, computed tomography, electron microscopy, and fluorescence microscopy. The input format was either two- or three-dimensional. With such a broad scope of challenges, Isensee et al. were able to achieve new state-of-the-art results in 33 out of 53 tasks and presented results comparable to the top of the leaderboard for the remaining 20 tasks.

In this research, preprocessing and parameter selection followed the standard nnU-Net approach, which selects all hyperparameters automatically. The method of selection differs depending on the type of parameters, which can be divided into the following three groups: fixed, rule-based, and empirical. Fixed parameters, i.e., the parameters that remain the same for all applications, include learning rate, loss function, architecture template, optimizer, data augmentation, training procedure, and inference procedure. The rule-based parameters, i.e., parameters selected based on dataset properties, include the following: intensity normalization, image target spacing, network topology, patch size, batch size, trigger of 3D U-Net Cascade, configuration of low-resolution 3D U-Net [27]. Some of the rule-based parameters are co-dependent, as they have to meet GPU memory constraints, e.g., image size affects patch size, which later limits batch size. Empirical rules, i.e., parameters that are tested during cross-validation, include configuration of postprocessing and ensemble selection. Key parameters selected for our task are listed in Table 2.

The nnU-Net framework explores 3 different U-Net configurations to find the best result. These 3 configurations include a 2D U-Net, 3D U-Net, and 3D U-Net Cascade. In case of the 2D U-Net, each slice of the 3D image is fed into the network separately. The 3D U-Net operates on full 3D images by cropping 3D patches that cover voxel space. The 3D U-Net Cascade operates in two stages. During the first stage, segmentations are made on a downsampled version of the image. In the second stage, these segmentations are improved using both the segmentation obtained in the first stage and the full-resolution image as input. Cascade is omitted if the 3D U-Net patch size can accommodate a large part of the input image.

Performance of each of the configurations is explored with the 5-fold cross-validation, during which the dataset is consistently split 5 times into training and validation sets and then tested on the test (unseen during the training) data. Based on the cross-validation results, nnU-Net compares the results of each of the configurations and ensembles of them and decides which performed best. The inference is performed by averaging over predictions from models trained during different folds of cross-validation. The sliding window method is used with a window size equal to the training patch size and Gaussian importance weighting to reduce stitching artifacts. The model selection as well as the inference are performed automatically and do not require additional user interaction [27].

Obtained training time was oscillating around 2 days per fold per model. Computations were run using 10 CPUs, a single GPU card Tesla V100 (Nvidia, Santa Clara, CA, USA). Version 1.7.0 of nnU-Net package was used for performing experiments. The Dice coefficient was used as the evaluation metric. The Dice coefficient, also known as Sørensen-Dice coefficient or F1 score, is one of the most commonly used evaluation and validation metrices in medical imaging machine learning segmentation tasks [28]. The formula for the Dice coefficient is as follows:

(1) $$\frac{2*TP}{2*TP+FP+FN}$$

where TP, FP, and FN represent the number of true positives, false positives, and false negatives, respectively. The Dice coefficient ranges from 0 to 1, with 1 meaning that two samples are identical, and 0 meaning that there are no mutual true positive data points.

In addition to being the standard metric for segmentation in medical imaging, the Dice coefficient was ideal for our application, as it is more focused on the total volume of the segmented lesion rather than a perfect reproduction of lesion borders [29]. As mentioned above, lymphoma borders could not always be identified in our dataset. What is more, the focus on volume reflects our suggested application of the model, which is comparison of segmentation volume in time.

Lymphoid volume for each test set segmentation was calculated using 3D Slicer software [24].

## 3. Results

The results of evaluating nnU-Net performance on each available model configuration can be found in Table 3. The highest Dice coefficient was obtained for the 3-D U-Net (without Cascade) model (0.7262 for the validation set). However, other model configurations achieved results that were comparable.

Table 4 presents the Dice coefficient for patients in the test set. The mean Dice coefficient for all the patients in the test set was 0.81 (SD = 0.17).

It should be noted that the model performed reasonably well on 4 out of 5 test cases, achieving a Dice score ranging from 0.73 to 0.95. One of the CT scans in the test set had a noticeably lower Dice score, i.e., 0.55. This was the result of the model interpreting part of the brain as a tumor-reference volume for this case was 146.19 cm^3^, and the predicted volume was 331.21 cm^3^. The volume of the mistakenly segmented brain tissue was larger than the reference volume of the tumor tissue, which translated to a low Dice score in this case. This was due to an unusually broad scanning range, and this is an example of the data coming from outside of the original distribution. Simple postprocessing by limiting the scanning range for that specific case would increase the Dice score greatly (to 0.92), but we refrained from that as the test set, in our opinion, should not be edited in any way to improve the results.

**Figure 2:**
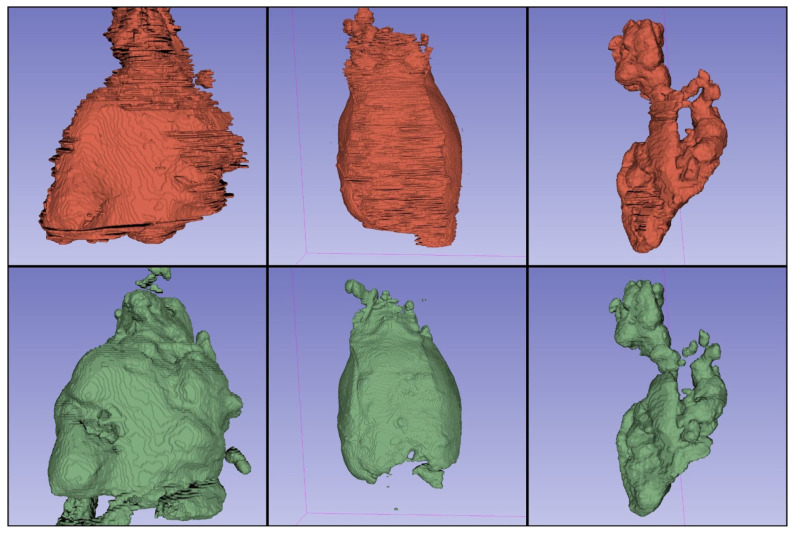
Three-dimensional renderings of the segmentations performed by a human reader (red) and from the best performing nnU-Net model (green). Smooth edge segmentation can be noted in the segmentations performed by the nnU-Net when compared to the human reader.

Exemplary segmentations are presented in Figure 2. It can be noticed that compared with manual segmentation, automatic segmentations have smoother borders. This reflects the challenges that are inherently associated with manual segmentation and clearly shows the potential advantage of machine learning-based approaches.

## 4. Discussion

Deep learning algorithms might be a solution to the growing number of imaging studies that greatly outpaces the number of radiologists being certified each year. With the aid of machine learning models, researchers develop ways of increasing the pace of reading imaging studies without compromising the quality, all the while reducing interobserver variability. For example, Lim et al. [30] recently published a study that applied deep learning algorithms for lumbar spine MRI assessment, which reduced reading time and improved the consistency of the stenosis rating between radiologists.

However, deep learning segmentation methods have their limitations that are, for the most part, independent of the research problem. Typical challenges include obtaining the ground truth segmentations, as this process is most often performed manually. This process is very time-consuming and may be prone to interobserver variability, as the borders of the lesions frequently cannot be discerned. Usually, a compromise needs to be made between the quality of the segmentation and the size of the dataset. In the following paragraphs, specific challenges encountered in our study and their consequences on our results are reported.

While evaluating the performance of nnU-Net on our dataset, some limitations were noticed that were mostly attributable to the limited size and diversity of the dataset. Firstly, there were cases of the algorithm mistaking parts of a solid organ, such as the brain or iliopsoas muscle, for tumor tissue. This issue occurred in patients with a broader than usual scanning scope. We believe this could be prevented by either including segmented whole-body CT scans from healthy individuals in the dataset or alternatively, by cropping the scope of the scans for existing cases to some standardized range. Although we are aware that not implementing these changes limits the use of our algorithm, it is beyond the scope of our project.

Another caveat of using human-made segmentations as the ground truth is that they can be quite far from the objective ground truth, in spite of best efforts. Perhaps the best illustration of the problem is presented by Tingelhoff et al. [31] as follows: in their experiment, 21 participants (10 ENT surgeons, 10 medical students, and one engineer) were asked to segment maxillary and ethmoid sinuses on the same CT scan. The total volume of the segmentation for the same patient varied between 30.9 cm^3^ and 47.1 cm^3^. This proves that the practical quality of even a relatively well-defined segmentation (as one would expect, would be the case for sinuses, being clearly limited by bones) is not as objective as it may first appear. What is more, manual segmentation is extremely time-consuming, and small improvements in quality may come with a significant increase in time per segmentation.

In addition to the ingrained inter-observer variability associated with any segmentation attempts, our team has encountered some further practical difficulties during the data preparation stage of the project, as described in the Materials and Methods section. This introduced further uncertainty to our human-made ground truth segmentation. The biggest challenges included the distinction between thymus and lymphoid tissue, as well as the presence of pericardial and pleural effusion and liquefactive necrosis within the tumor. However, it must be noted that even experienced radiologists might find the differentiation difficult while reviewing computed tomography. These issues translate into the model handling poorly some edge cases during cross-validation. Depending on the fold (and, therefore, on the training set subset), the model tended to be more likely to include or exclude effusion, liquefactive necrosis, and normal thymus in the automatic segmentation. Nonetheless, as the output of our model consists of voxel-wise segmentations, it can be edited by a reviewing radiologist to exclude any regions that were erroneously included. Providing editable, three-dimensional output segmentations might provide at least a partial solution to this problem.

It is important to underline that other researchers encounter similar difficulties. However, the positive impact of publishing imperfect training datasets on which other researchers can advance their research is worth emphasizing and promoting.

Our dataset included 30 CT scans from 30 different patients. To the best of our knowledge, all the previously published studies attempting automatic lymph node segmentation were also performed on small datasets, with the notable exception of the paper by Roth et al. [32], who published a large dataset of 176 abdominal CT scans with segmented lymph nodes. However, on manual inspection, it could be noted that many of the lymph nodes in the region of interest were not segmented, which is understandable, given the size of the dataset and the fact that, on average, there are 230–250 abdominal and pelvic lymph nodes [33]. To the best of our knowledge, there is no publicly available large dataset of segmented CT scans of patients with lymphoma. It can be hypothesized that the reason for this is a very large time investment on behalf of scientists attempting to create such a dataset, as one case may take several hours to segment. While it is clear that the scientific community would greatly benefit from large, publicly available, high-quality datasets with segmentations of various tissues, this project attempted to create a valuable dataset that would enable to train a reasonably well-performing model under practical constraints. It should be underlined that increasing the size of the dataset could decrease the variance in performance on the test set (including the model interpreting part of the brain tissue as a tumor, as seen on Patient 4 of the test set), and therefore, contribute to its overall better performance. What is more, the lack of appropriate publicly available datasets made it impossible to perform external validation on our model. We hope international scientific cooperation can help to alleviate some of these problems in the future.

In spite of being generally performed on small datasets, multiple attempts of automatic lymph node segmentation on CT scans have been reported. Early attempts employed numerical methods analyzing the shape, gray value, and borders of the nodes that generally required placement of a marker inside a lymph node that was to be segmented [34], although some models were also able to detect lymph nodes [35] without placing the marker. More recently, Iuga et al. [36] proposed a CNN for the detection and segmentation of thoracic lymph nodes in patients with possible lymph nodes metastasis. However, their approach differed from ours, as multiple small, non-pathological lymph nodes were generally assessed, and their primary benchmark was the number of detected lymph nodes and not their volume. The total detection rate was 69.9% for the validation dataset.

The development of new deep learning architectures has allowed a significant advancement in many applications of machine learning in medical imaging. For this project, the nnU-Net framework was used. This decision was based not only on the application of a well-designed deep learning model but also on a toolkit for pre-processing of the data, increasing the efficacy of hardware use, training process, cross-validation, supporting reproducibility, and more. Good deep learning practices that are an integral part of the nnU-Net guarantee that many common errors will be avoided and resulting models will be of high quality.

## 5. Conclusions

In this paper, a novel approach to a potentially important clinical task—volume calculation of thoracic lymphoma in pediatric patients—is presented. As discussed in the introduction, lymphoma volume correlates well with disease prognosis; therefore, accurate evaluation directly affects clinical management. We suggest that such tasks should be fully automated using recent advancements in imaging processing with machine learning methods. This study proves the feasibility of this approach and can serve as a building block for the further development of fine-tuned methods. It should be emphasized that there are some limitations of this study, including the size of the dataset and lack of external validation, both connected with the lack of public datasets. The nnU-Net, which was used for this project, is a freely available software package. The dataset used for the training and evaluation is published as a supplementary material with open access, and we encourage other researchers to use and modify it. To our best knowledge, it is the first, open, labeled dataset for pediatric lymphoma segmentation. As pointed out by Varoquaux et al. [37], dataset availability greatly influences areas of focus in a given field of research. Therefore, we expect other researchers to use this dataset freely to develop new machine learning algorithms, which will eventually bring benefit to the patients affected by this condition.

What is more, further prospective studies verifying the practical value of calculating the total tumor volume are needed, and the relationship between total volume change and clinical progression/remission should be documented.

## Figures and Tables

**Table 1:** Challenges encountered by the radiologists during segmentation and solutions used in the database.

Region/Issue	Solution
Cervical lymph nodes not always distinguishable from surrounding tissues	Include cervical lymph nodes whenever possible
Unsharp border between lymphoma and thymic tissue	Exclude thymus from segmentation only when a clear border between lymphoma and thymus is visible; include thymus in segmentation when no clear border is visible
Unsharp borders between lymphoma/liquefactive necrosis and fluid in pericardium and pleural cavities	Try to exclude any pericardial and pleural effusion and include liquefactive necrosis in the segmentation (difficult in some cases)
Abdominal lymph nodes	Do not include in the segmentation

**Table 2:** Key parameters of the nnU-Net framework. SGD-stochastic gradient descent.

Parameter	Value
Batch size	2D: 12
	3D: 2
Float precision 16-bit	Yes
Max number of epochs *	1000
Number of batches per epoch *	250
Number of input channels	1
Initial learning rate *	0.01
Momentum *	0.99
Optimizer *	SGD
Patch size	2D: 512 × 512
	3D: 96 × 160 × 160
Weight decay *	0.00003

* fixed parameters.

**Table 3:** Model performance measured by the average Dice coefficient during cross-validation. The model with the highest dice score is highlighted.

Model	Average Dice Coefficient
2D U-Net	0.7065
3D U-Net	0.7262
3D U-Net Cascade	0.7024
2D U-Net + 3D U-Net	0.7221
2D U-Net + 3D U-Net Cascade	0.7203
3D U-Net + 3D U-Net Cascade	0.7148

**Table 4:** The test set results for the 3D U-Net model. The dice coefficient, reference volume based on the manual segmentation, volume predicted by the model, and volume difference are presented for each patient.

Patient	Dice	Manual Segmentation [cm^3^]	Automatic Segmentation [cm^3^]	Volume Difference [cm^3^]
Patient 1	0.88	288.79	257.68	31.11
Patient 2	0.73	631.34	865.01	−233.67
Patient 3	0.92	776.99	686.14	90.85
Patient 4	0.55	146.19	331.21	−185.02
Patient 5	0.95	354.63	352.09	2.54

## Data Availability

The data presented in this study are openly available in Zenodo at [21,22].
